# Opium and Quinia Sulpatis

**Published:** 1864-12

**Authors:** T. D. Fitch

**Affiliations:** Chicago


ARTICLE XLII.

### OPIUM AND QUINIA SULPATIS. THEIR ANTAGONISM AND CO-OPERATION.

BY T. D. FITCH, M.D., of Chicago.

Read before the Chicago Medical Society.

Dr. GUBLER says, in a French Journal of Pharmacy, that they are antidotes to each other, and that they ought not to be administered simultaneously. Dr. GUBLER is not alone in this decision. Many others have, from observation, arrived at the same conclusion.

We assume that they are not antidotes to each other, only so far as relates to their bad or undesirable effects; and that they may be administered simultaneously, obtaining all their valuable effects when given alone, and under some circumstances, that their reaction upon each other produces the most happy therapeutic results.

Dr. GUBLER says that, the effects of quinia on the circulation are inversely to those of opium. It gives tone or contractility to the capillaries, and thus overcomes congestion. It is antiperiodic and sedative. Opium is a stimulant to the capillary system, and produces congestion, especially of the brain and lungs. Now this is the very point we wish to touch. Quinia, to a great extent, antidotes these bad effects. Being a tonic to the capillary system of vessels protects them from serious accumulations by giving additional tone and contractility.

Dr. CORRIGAN, physician in ordinary to the Queen, in Ireland, says, quinia appears to possess the same power in giving contractility to the capilliaries in the lungs, which we know it possesses in so marked a degree over the capillaries and venous radicles of the spleen. This property of quinia gives a power over almost all forms of venous and capillary congestion, which perhaps it is impossible to obtain by any other known agent.

Opium when given alone in inflammatory affections, is liable to so far paralyze the nervous energies, as to produce fatal congestions. We have seen that quinia possesses the power of producing capillary contraction and tonicity, and so keeping up the tone of the vessels as to completely prevent any such paralysis and its consequent congestion, so that the full effect of opium may be obtained without the least danger of this result.

Opium has the effect, when given alone, of so far diminishing the billiary and renal secretions as to endanger fatal toxemia. It also diminishes all mucous secretions, salivary and pancreatic secretions. It also produces constipation, partly by the check to the secretion from the liver, and partly by directly paralyzing the muscular coats of the bowels. These unpleasant properties and effects render the use of it objectionable in fevers, inflammations, &c., where, as a general thing, these secretions are already deficient, and the bowels constipated. Could these bad effects be avoided, there are many important indications in those complaints that could be most perfectly filled by the use of opium. Now quinia combined with the opium will, to a very great extent, prevent all those bad effects, while it does not materially diminish its action on the skin. In fact, I believe that its diaphoretic properties are increased rather than diminished.

On the lungs its operation is likewise modified. When given in large doses, it has the effect to so far reduce the number of respiration per minute, that it materially interferes with the proper oxygenation of the blood; as we have seen by the remark of Dr. Corrigan, quinia has peculiar power over the capillaries of the lungs, thereby preventing fatal congestion, and keeping the number of respirations to a more healthy standard.

The after effects of opium, we are all familliar with, such as nausea and vomiting, severe headache, general prostration, &c. All physicians are perplexed with such results, which nearly or quite counterbalance all good that has been experienced. We therefore resort to other preparations of the drug, hoping to find something that will be borne without such unpleasant results, morphia, sulph., mur. and acet., black drop, McMun's elixir fluid extract, and sometimes having to content ourselves with the use of an inefficient substitute, such as hyoscyamus, belladonna, cicuta, and lupulin, none of them filling all the indications filled by opium when well borne.

The foregoing unpleasant symptoms seldom if ever make their appearance when opium is associated with quinia. The prickling and itching sensations felt in the skin, and especially the nose, are not at all modified by the use of quinia, it having no antidotal effect on the diaphoretic properties of opium.

Many persons cannot take opium on account of peculiarity of temperament, or idiosyncrasy. In these cases the manifestations are variable, delirium and great restlessness; and in these cases quinia given in combination with the opium produces the happiest effects; in fact, I have often seen this exemplified in my own practice. I have never paid any attention to this idiosyncrasy since I commenced giving these two remedies combined. I have administered them to many persons that could not take opium at all in former years, without the least unpleasant effect.

In cases of extreme exhaustion, after protracted hemorrhages, opium is known to be one of the best stimulants to the circulation. The system then tolerates the remedy most wonderfully; but in these cases, when reaction does come on, there may be sufficient opium yet in the system to produce serious if not fatal narcotism. Quinia will enable us to keep up the vital forces without the use of opium so frequently or so largely. By the foregoing it will be seen that most of the unpleasant effects of opium may be entirely relieved by the association of quinia.

On the other hand, the unpleasant effects of quinia, such as ringing in the ears, and noises in the head, always produced by large doses of quinia, are seldom if ever experienced when opium is associated with it. The nausea and vomiting, and general constitutional irritation so frequently produced where quinia is given alone, is seldom if ever produced when opium is added.

In many cases where the well known effects of quinia are very desirable, and peculiar circumstances prevents its administration, for instance, in active inflammations or fevers, we frequently get an excitement and general increase of fever and of the circulation. Opium combined with quinia under such circumstances, relieves irritation—makes a diversion of the circulation to the skin, producing free perspiration, so that the quinia may be tolerated in large doses, and its peculiar effect on the capillaries and nerves secured.

In neuralgias and rheumatisms, where both remedies are acknowledged of great value, when given alone, their combined action is found generally to be much more satisfactory. The opium relieving irritation and pain, diverting the circulation to the skin, producing copious perspiration, and in fact, generally equalizing the circulation, while the quinia acts as a permanent tonic, and anti-periodic where malarial poison exists in the system.

In acute catarrh, coryza, or general cold, fever, pain in the back, extremities, and frontal sinusses, the patient may be directed to take quinia sulph. grs. v. to viij., opii. pulv. grs. ij. to iij. every three hours, (after an active cathartic has been given, where the bowels are constipated), and the result is that after one or two doses have been taken, free perspiration follows with entire freedom from all pain, and the patient soon recovers.

This combination is especially useful in dysentery, after the bowels have been properly evacuated, and the secretions attended to by the administration of proper alteratives.

Its effects are more striking, if possible, in idiopathic or malarial fevers, when the effects of quinia are indispensable; in these cases it may be given, after proper alterative and evacuative treatment has been instituted, in any stage, whether in presence or absence of fever, and a perfect intermission in the fever procured in most cases.

My practice in these cases is to administer when the bowels are constipated an active alterative cathartic, then taking the most favorable time, which is always in the morning, I give, say, three powders, three hours apart, composed each of quinia sulph. grs. v. to viij., opii. pulv. grs. ij. to iij., the effect is to produce free diaphoresis and allaying of all pain, a lessening of the frequency and force of the pulse, and the mucous secretions are more abundant. I then omit the remedy, following with such simple treatment as the case requires, until the next morning, when I repeat the process with like results. In from one to three days a perfect intermission is secured, and what might have resulted in a long continued or typhoid fever, is converted into a simple intermittent, or entirely interrupted and completely cured. The combined action of these two remedies in these cases, I consider a desideratum, and worthy a trial by those of you who have not already witnessed their effects.

If these are facts which I have stated, you will readily see that these two remedies are not essentially antagonists but a co-operatives, and applicable in the treatment of a large class of cases which we meet in this country.

